# Influence of Citric Acid-Assisted Impregnation of Recycled Aggregate on the Properties of the Resultant Concrete

**DOI:** 10.3390/ma16082986

**Published:** 2023-04-09

**Authors:** Roman Jaskulski, Wojciech Kubissa, Yaroslav Yakymechko

**Affiliations:** 1 Department of Civil Engineering, Wrocław University of Environmental and Life Sciences, 50-375 Wroclaw, Poland; 2 Faculty of Civil Engineering Mechanics and Petrochemistry, Warsaw University of Technology, 09-402 Plock, Poland; 3 Faculty of Civil Engineering, Cracow University of Technology, 31-155 Cracow, Poland

**Keywords:** recycled concrete aggregate, impregnation, citric acid, water glass, calcium hydroxide

## Abstract

The paper presents the results of tests on concrete with recycled aggregate impregnated with the use of citric acid. Impregnation was carried out in two stages, with a suspension of calcium hydroxide in water (so-called milk of lime) or diluted water glass used as the second impregnant. The mechanical properties of the concrete were carried out: compressive strength, tensile strength and resistance to cyclic freezing. In addition, concrete durability parameters such as water absorption, sorptivity and torrent air permeability were investigated. The tests showed that this type of impregnation did not improve most of the parameters of concrete with impregnated recycled aggregate. The mechanical parameters after 28 days were significantly lower compared to the reference concrete, although after a longer curing period, these differences decreased significantly for some series. The durability parameters of the concrete with impregnated recycled aggregate also deteriorated compared to the reference concrete with the exception of air permeability. The results of the tests carried out indicate that impregnation using water glass in combination with citric acid gives the best results in most cases and that the order in which the impregnation solutions are applied is very important. Tests also showed that the effectiveness of impregnation is very much influenced by the value of the w/c ratio.

## 1. Introduction

Depleting natural resources of raw materials, combined with increasing environmental concerns, are creating growing pressure to reduce landfill and reuse waste. This trend also applies to the construction industry, which is a major consumer of raw materials and producer of waste from, among other things, the demolition of various types of structures [1,2,3,4]. While the reuse of steel recovered from construction is almost complete and follows technology that has been used for many decades in the metallurgical industry, waste from the demolition of concrete structures is still reused in far too small a quantity.

One obstacle is the deterioration in concrete performance usually obtained when replacing natural aggregate (NA) with recycled concrete aggregate (RCA) in concrete of the same composition: lower strength, higher water absorption and thus lower resistance to environmental corrosion. The deterioration of the properties of concrete with recycled aggregates can be counteracted in various ways with two approaches being the most common. The first involves enriching the composition with supplementary cementitious materials [5,6,7]. This is usually fly ash, ground granulated blast-furnace slag, silica fume or metakaolin [8,9,10,11,12,13,14]. The second approach focuses on the aggregate itself and its treatment before use in concrete.

Methods for treating recycled concrete aggregate are manifold, and new ones are still being proposed. They can be divided into two groups [15,16,17]. The first group includes methods aimed at removing as much as possible the old mortar adhering to the grains of the original natural aggregate. These methods are divided into mechanical, involving grinding of the aggregate [18,19] including grinding with prior heating [20,21], and chemical, in which acids are used to remove old mortar [22,23]. However, they all have the disadvantage that they result in further waste that has to be managed. In the second group are methods that aim to strengthen the old mortar and improve its properties. Among these methods, impregnation is the most common, using sodium silicate solutions [19,24,25], lithium silicate [26], PVA [25,27], silane [24,25], silica fume and nano-silica [28,29], cement slurry [30] and cement with silica fume [19]. The possibility of sealing recycled aggregate by depositing calcium carbonate in the pores of old mortar is also being investigated [31,32], as well as improving its properties by ultrasonic washing [28]. The effects of combining these methods from both groups, i.e., first removing the old mortar and then improving the pre-treated aggregate, are also being investigated [33].

When reviewing the literature, it is easy to see that there are relatively few descriptions of studies in which RCA is impregnated in two stages, and virtually none in which one of the impregnants is citric acid. It is relatively rare in the literature to find results of studies of such durability characteristics of concrete with impregnated RCA as resistance to cyclic freezing or air permeability. Taking this into account, this paper fills the knowledge gap on the effect of two-stage impregnation of recycled concrete aggregate with the use of citric acid on the mechanical and durability properties of concrete, with particular emphasis on permeability tested by the Torrent method.

The research presented in this article is a continuation of investigations presented in [30,34]. As in those studies, recycled concrete aggregate was soaked in various solutions. As in [34], the impregnation was carried out in two stages. However, while in the previous studies the aim was to reinforce the old mortar, different assumptions were made for part of the series in the currently described studies.

A total of 10 series of concrete with varying w/c values was made, 8 of which contained recycled concrete aggregate subjected to a two-stage impregnation procedure. One of the impregnants was a citric acid solution applied to the aggregate in all eight series.

The citric acid used had a twofold function. The first was to remove some of the old mortar, and for this purpose it was used in the first impregnation step. Then, the goal of the second impregnation was to strengthen the mortar that had not been removed. The use of citric acid in the second impregnation step, on the other hand, was intended to have a synergistic effect with the first impregnation and lead to an improvement in the properties of the old mortar.

Tests of selected mechanical and durability properties of concrete were used to assess the achievement of the intended effects. These effects have been only partially achieved, and the reasons for this are discussed later in this paper. Furthermore, although the results obtained are not very promising, the critical analysis of the research carried out in the paper provides grounds to conclude that with some adjustments to the impregnation procedure, the effectiveness of the presented methods of treatment of recycled concrete aggregate can be proven. It also gives guidance to the direction in which the modification of the presented procedures should go.

## 2. Materials and Methods

### 2.1. Materials

Ten series of concrete were made for the study. Five of them were made assuming w/c = 0.45, and another five assuming w/c = 0.55. The concrete series with the same w/c value differed from each other only in the type of aggregate used and the amount of superplasticizer added to the mix. The aggregate was obtained by crushing the concrete in a laboratory jaw crusher. The concrete came from cubes left over from the strength test and was at least two years old at the time of crushing but not exceeding five years. The compressive strength of the crushed concrete varied and was approximately in the range of 20–40 MPa. The choice of such a raw material for the preparation of recycled concrete aggregate was dictated primarily by its availability. It was also significant that it was thus possible to guarantee a large diversity of source concrete in terms of strength, type of natural aggregate used, type of cement and age. The use of aggregate from the demolition of a particular structure would not have yielded the variety of raw material that positively influenced the versatility of the results obtained.

The preparation of the aggregate involved several steps carried out in two stages. In the first, raw recycled aggregate was obtained, and this stage included pre-cutting of the concrete pieces, soaking (7–14 days), crushing, primary screening to separate oversize and sub-grains, crushing of oversize grains (>16 mm), screening by fractions (2–4 mm and 4–16 mm) and mixing of individual aggregate batches. In the second stage, the portion of aggregate to be used in specific series of concrete was twice impregnated and dried.

Due to the amount of aggregate required, it was prepared in smaller portions. Because of this, as well as due to the random selection of the portions of material for subsequent operations, the aggregate obtained was homogenized to a large extent. Consequently, it could be considered that the initial variation in the parameters of the concrete serving as raw material would not affect the significant variation in the characteristics of the aggregate obtained.

**Figure 1:**
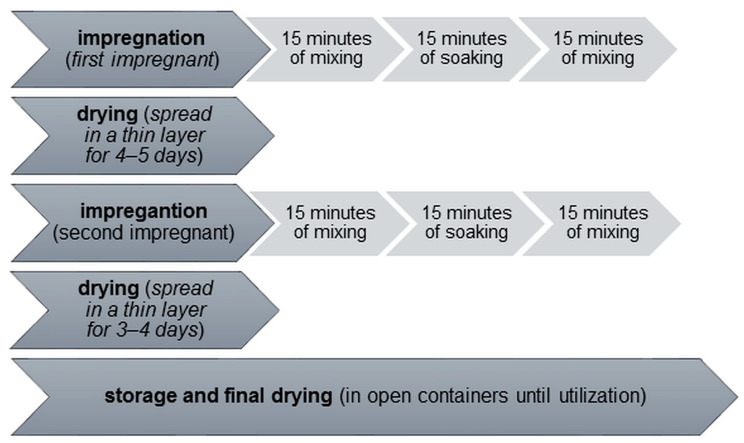
Flowchart of impregnation process.

The second stage of preparation was double impregnation. Aggregates intended for a given batch of concrete were mixed with the impregnating solution for 30 min in a slow mixer—twice for 15 min each. There was a break between the first and second mixing, also lasting 15 min. Once the impregnation was complete, the excess impregnating solution, if any remained, was separated from the aggregate, and the aggregate itself was spread out in as thin a layer as possible to dry in the laboratory. After the first impregnation stage, the aggregate was left to dry for 4–5 days, after which a second impregnation was carried out. After the second stage, the aggregate, after drying for 3–4 days in a spread form, was transferred to open containers, where it was stored until it was used. The second impregnation stage took place at least 14 days before concreting. The flowchart of the impregnation process is presented in Figure 1.

Three types of impregnants were used for treatment. They were applied in different combinations and in different orders. These were as follows: 5% citric acid solution, which was used in each case, and interchangeably lime milk, which is a suspension of calcium hydroxide in water (1 part Ca(OH)_2_ to 2 parts water) and diluted water glass (1 part water glass to 2 parts water). The compositions of all impregnants were determined by weight. The impregnating solutions used and their order of application are reflected in the series designations. The first letter in the series name indicates the first impregnant used (C—citric acid, L—lime milk and G—glasswater), the second letter indicates the second impregnant, and the number indicates the w/c value (4 indicates w/c = 0.45, and 5 indicates w/c = 0.55).

In the two reference series, the aggregate was not impregnated, but in order to maintain similar conditions of preparation, it was washed with water and then dried. The concrete reference series were designated by the letters NI. These series used recycled concrete aggregate that was not impregnated. The compositions of all the series made are shown in Table 1.

In each series, 36 cubic specimens with a 100 mm edge and 2 cubic specimens with a 150 mm edge were prepared for testing. Due to the high content of unreacted citric acid in the aggregate, which is a compound that strongly slows down cement setting [35,36,37], the specimens could only be unmolded after 7 days to minimize the risk of damage. Until this time, the specimens were in the molds covered with foil. After demolding, the specimens were stored in water until testing with the exception of the 150 mm edge cubes, which were removed from the water 28 days after concreting and stored in the laboratory under air-dry conditions until the testing procedure started.

### 2.2. Methods

Compressive strength (after 28 days and 36 weeks), tensile strength (after 28 days), water absorption, sorptivity and freeze–thaw resistance were tested on specimens prepared from each concrete mix.

The compressive strength after 28 days was tested according to the procedure given in EN-12390-3 [38] on 6 cubic specimens with an edge of 100 mm. The specimens were stored in water until tested. The compressive strength after 37 weeks was tested according to the same procedure, but on two 150 mm-edge cubic specimens, which had previously undergone an air permeability test. As the permeability measurement is a non-destructive test, so the results obtained on these specimens can be considered representative and allow for comparison of the properties of the prepared concrete series after a longer period of time. The specimens in this study were in a dry state, as they were subjected to cyclic drying as part of the air permeability test procedure described later in this chapter.

The tensile splitting strength was tested according to the procedure described in EN-12390-6 [39] on six cubic specimens with an edge of 100 mm. The specimens were stored in water until the test. The specimen halves obtained from the test were then used to carry out water absorbability and sorptivity tests. 

In order to determine the water absorbability, the 12 halves of the cubic specimens from each series remaining after the splitting tensile test were placed in water for a further 7 days. After this time, they were removed, surface-dried and weighed. The mass thus determined was taken as the mass of the specimen fully saturated with water (m_s_). The weighed specimens were placed in a forced-air dryer at 110 ± 5 °C. After 14 days of drying, the specimens were removed and weighed, taking the mass thus determined as the mass of the dry sample (m_d_). From these two mass values, the absorbability N was determined according to Formula (1):

N = (m_s_ − m_d_)/m_d_·100%. (1)

The dried specimens after the water absorbability test were left to cool after the weighing. The sorptivity of the concrete was then tested on them using the mass method. The test procedure used in this case is described in detail in [40], as is the method of calculating the sorptivity from the results of successive weighing of the 12 cubic specimen halves tested. The method is similar to that given in ASTM C1585 [41], but the lateral surfaces of the specimens were not sealed, and the specimens themselves were dried to a constant mass. 

**Figure 2:**
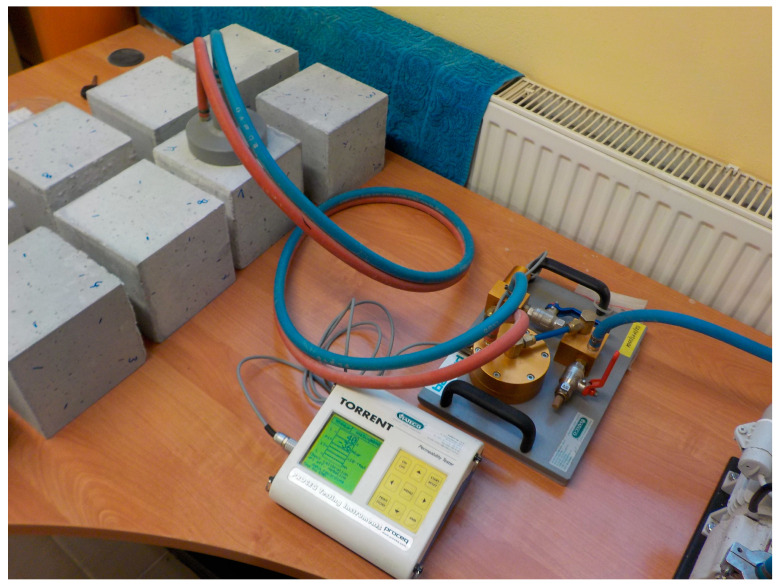
Torrent air permeability of concrete testing.

The air permeability of the concrete was tested using a Torrent apparatus manufactured by Proceq. Two cubic specimens with an edge of 150 mm were used for the test. Measurements were taken on the lateral surfaces, i.e., perpendicular to the direction of concreting. The probe of the device was applied approximately in the middle of the wall at a distance of at least 20 mm from the edge. In total, permeability was measured on eight specimen surfaces for each batch of concrete. An example of a Torrent test is shown in Figure 2.

Prior to application of the Torrent apparatus probe, the moisture content of the surface layer of concrete was determined with a meter using low-frequency current impedance measurement. To increase the accuracy of the moisture measurement, the meter was applied to the specimen four times on each wall, each time changing its orientation relative to the surface to be measured by rotation. 

The concrete permeability tests for this article were carried out as part of a longer procedure to investigate the dependence of this parameter on the moisture content of the material. As part of this procedure, the specimens were immersed in water for a fortnight prior to testing. They were then left to dry in the laboratory under air-dry conditions for a further 12 days. Since, according to Torrent [42], testing the permeability of concrete for the purpose of determining its quality should be carried out at a moisture content of less than 5.5%; this article reports the results from the second cycle of measurements. In the first cycle, the moisture content of the majority of the specimens exceeded the specified threshold value. Between the first and second measurement cycles, the specimens were dried at 65 °C for 4 days and then seasoned under laboratory conditions for a further 6 days. As a result of this procedure, the moisture content of the specimens in the second test cycle was below 5%.

A freeze–thaw resistance test of concrete was carried out using 12 cubic specimens with an edge of 100 mm each. The specimens were stored in water from the time of concreting until the test. For the duration of the test, 6 specimens were placed in a freeze–thaw test chamber and subjected to 100 freeze–thaw cycles, while the remaining 6 were left in water as so-called specimen witnesses. The test cycles were carried out according to the principles of the so-called ordinary method described in the Polish standard PN-B-06265 [43]. The specimens were frozen in air at −18 ± 2 °C for 4 h. The thawing of the specimens took place in water at 18 ± 2 °C for 2 h. After the final cycle, the specimens from the test chamber were transferred to a container with water and then subjected to a compressive strength test. Specimens not subjected to cyclic freezing/thawing were also tested at the same time. The result of the test was the ratio of the compressive strength of the frozen specimens to the specimen witnesses.

Independently of the freeze–thaw resistance test according to Polish standard PN-B-06265 [43], a test was also planned to determine the decrease in tensile splitting strength as a result of cyclic freezing–thawing. The test was carried out according to the same assumptions, taking into account only the differences resulting from the different strength testing procedure.

## 3. Results

The results of the tests carried out, apart from the freeze–thaw resistance test, are given in Table 2. The representative values of each of the measured parameters are expressed by the following: the median of the results obtained in the case of compressive strength after 28 days, tensile strength, water absorbability and sorptivity. In the case of air permeability coefficient, the geometric mean of the results was calculated, and in the case of concrete moisture content and strength after 37 weeks, it was the arithmetic mean.

The median absolute deviation (MAD) is a measure of the variability of the individual parameters for compressive strength after 28 days, tensile strength, water absorbability and sorptivity. In the case of concrete moisture content, the standard deviation of the results was calculated. As the compressive strength value of the concrete after 37 weeks was only tested on two specimens, the spread, i.e., the difference between the two values, was given in this case.

For the air permeability coefficient, it was decided to define its own measure of variability, which was defined as the average geometric deviation γ, and calculated according to Formula (2):

γ = G_ω_ (2)

where γ—average geometric deviation, G_ω_—geometric mean of the multiples of the mean ω_i_ calculated according to Formula (3):

ω_i_ = x_i_/G_x_ if x_i_ > G_x_ or ω_i_ = G_x_/x_i_ if x_i_ < G_x_ (3)

in which x_i_—the value of a single measurement, G_x_—the geometric mean of all measurements. In the case where the value of ω_i_ satisfies the inequality (4), then the result to which the calculated value of ω_i_ corresponds was considered an outlier, and the value of γ was recalculated with this result omitted.

ω_i_ > 3G_ω_ (4)

The results of the freeze–thaw resistance test are shown in Table 3. The median and median absolute deviation values of the compressive strength of specimens subjected to freeze–thaw cycles and specimen witnesses stored in water during the tests are given. From the median values, the mean decrease in compressive strength of concrete subjected to freeze–thaw cycles was calculated. In cases when the specimens were damaged during freeze–thaw cycles, only the median strength of the witness specimens is given in Table 3. For the tensile splitting strength drop test, the results of specimens subjected to cyclic freeze–thaw were obtained for only four series.

## 4. Discussion

An analysis of the results obtained leads to the conclusion that the impregnation of the aggregate carried out did not improve the tested parameters of the concrete made. That is the most general conclusion, but a closer look at the obtained results of individual tests allows one to indicate cases in which there was, however, a slight improvement in the parameters. A closer analysis also makes it possible to indicate which of the solutions adopted have no prospects of success and which offer a chance of improving the properties of the concrete after some changes in the assumptions.

The reason for the failure is undoubtedly citric acid—not, however, its use per se, but the assumptions made in doing so, most notably the amount of it remaining on the aggregate grains after the impregnation was completed. As written earlier, this compound was intended to play one of two roles in the mixtures being prepared. Either it was to remove some of the mortar from the recycled concrete aggregate, or it was to enhance the action of one of the other impregnations due to chemical reactions between both impregnations. It appears that the effectiveness of citric acid in both roles depended on which other impregnate it was used with. However, the effects of the impregnation were also influenced by the value of the w/c ratio of the concrete with the modified aggregate. The addition of citric acid did not work particularly well in combination with lime milk and also performed less well as a second impregnator. The combination with glasswater as a second impregnant yielded better results. In this combination, the order of application of the impregnating solutions was definitely more important for concrete with w/c = 0.55.

As far as the impregnant combination is concerned, there are two reasons why citric acid and glasswater work better together. The first is the reaction products. When the acid reacts with glasswater, its product is a silicone gel [44]. Its structure and hardness depend on the concentration of the reactants. In its structure, unless it is washed away with water, there is a second product of the reaction, namely, the sodium salt of citric acid [45]. It may affect the hydration of the cement, but the key point is that the reaction that occurs produces a solid product bound to the aggregate particles.

The second reason is the practically unlimited solubility of glasswater in water. This allows glasswater to be deposited in the pores of the aggregate in the first stage of impregnation in an amount that depends primarily on the concentration of the solution used and the available pore volume. At the same time, due to the high viscosity of glasswater itself, it is likely that the concentration dependence is not monotonic but has an extreme in its course. 

Lime milk, in contrast to glasswater, is very poorly soluble in water. Its solubility at 20 °C is 1.27–1.35 g/L, depending on particle size and dosage [46]. This is therefore an upper limit on the amount of Ca(OH)_2_ that can be deposited in the pores of the aggregate per unit volume by introducing it as a solution. This is the reason why lime milk was used instead of a saturated solution of Ca(OH)_2_. The aim was to increase the amount of this compound deposited in the aggregate, although mainly on its surface.

The reaction of Ca(OH)_2_ with citric acid produces calcium citrate, whose solubility at 20 °C ranges from 3.91 mmol/L (hexahydrate) to 5.69 mmol/L (tetrahydrate) [47], which translates into 2.37 and 3.25 g/L, respectively. The compound crystallizes as a loose precipitate. This precipitate was intended to tighten the aggregate structure by crystallizing in the pores. Judging by the effects, it can be assumed that if the first impregnation solution was lime milk, the amount of Ca(OH)_2_ deposited in the pores of the aggregate was so small that the citrate precipitate formed by the reaction with citric acid had little effect on sealing the pores.

Moreover, the neutralizing role of Ca(OH)_2_ towards citric acid was also low with this sequence of the impregnation. This was unlike the case when citric acid was the first impregnating solution. In this case, the low solubility of Ca(OH)_2_ was not a hindrance, because as the calcium citrate crystallized, another portion of calcium hydroxide was dissolved in the water. This reaction continued as long as there were reagents available and water as the reaction environment. It can be assumed that this was still happening while the aggregate was still drying. 

The order of impregnation was also important when using water glass. It should be noted that during the second stage of impregnation, when there is an interaction between the two compounds contained in the impregnating solutions, the second impregnant is always in excess of the first. This is because there is only as much of the first as the aggregate has accepted. In this way, the compound contained in the second impregnating solution gives the aggregate its “character”, including determining its pH value. This has consequences during the setting and hardening of the concrete mixture made with the impregnated aggregate.

### 4.1. Results of Compressive Strength Test

**Figure 3:**
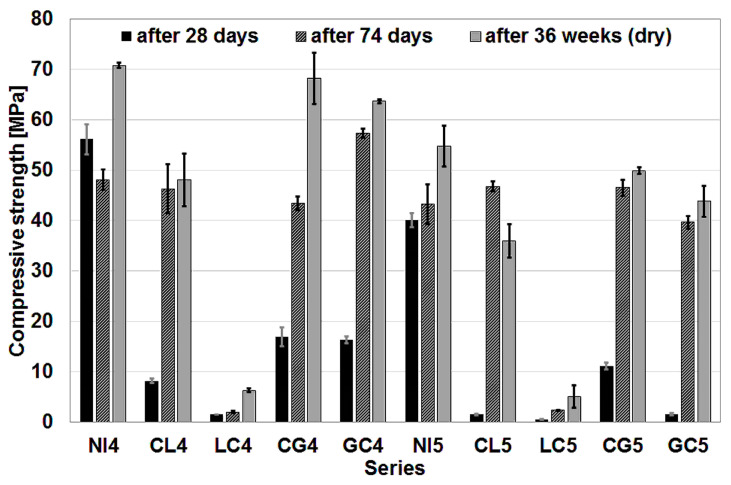
Results of the compressive strength tests.

Figure 3 shows the results of the compressive strength tests. The results of tests carried out after 28 days are placed first. They clearly show that the impregnation worsened the compressive strength of the concrete measured after the material had matured for 4 weeks. Comparing only the series with the impregnated aggregate between each other, it is worth noting that the best result was obtained for concrete with w/c = 0.45 and aggregate impregnated first with citric acid and then with water glass. At this w/c value, changing the order of application of the impregnating solutions slightly reduced the strength of the concrete. On the other hand, adopting w/c = 0.55 resulted in lower strengths, with a very significant drop in strength when the aggregate was impregnated first with glasswater. 

A slightly different picture emerges if the results obtained after 74 days are analyzed. The compressive strength after such a time was tested on the witness specimens from the cyclic freeze–thaw test. If the series with w/c = 0.45 are analyzed, in one case, the GC4 series, the obtained strength value of the concrete was higher than in the case of the reference series. The difference was very pronounced and certainly significant. The use of impregnated aggregate in concrete with w/c = 0.55 resulted in an increase in strength compared to the reference concrete in two cases: the CL5 and CG5 series. In all other cases, a decrease in compressive strength was recorded. Irrespective of the w/c value, concrete with aggregate impregnated first with citric acid and then with lime milk achieved quite similar strengths as did concrete with untreated aggregate, namely, once slightly higher and once lower. The reverse order of impregnation resulted in concrete with very low strength.

The results of the test after 36 weeks, carried out on dried specimens with larger dimensions (150 mm × 150 mm × 150 mm), showed a slightly different relationship between the strength values obtained for the different series. Irrespective of the value of the w/c ratio, the highest strengths in these tests were obtained for the reference series. This was followed by series with aggregate impregnated first with citric acid and then with glasswater. Slightly lower strength values were obtained by series with aggregate that was impregnated with the same solutions but in reverse order. Concrete with aggregate impregnated first with citric acid and then with lime milk obtained significantly lower strengths than the previously mentioned series, but these were still acceptable values, which cannot be said about the results of the concrete in which the aggregate was impregnated first with lime milk and then with citric acid.

### 4.2. Results of Tensile Splitting Strength Test

**Figure 4:**
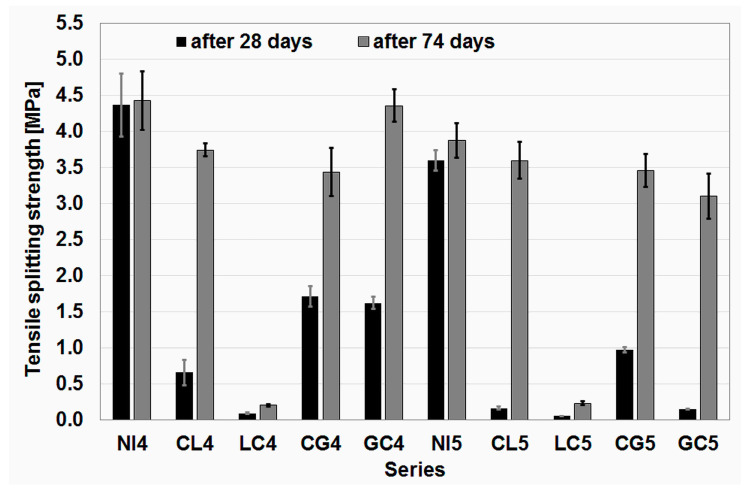
Results of the tensile splitting strength tests.

The results of the tensile splitting strength test after 28 days and 74 days are presented in Figure 4. The results from the later date were obtained by testing the witness specimens in the tensile splitting strength drop test procedure as a result of cyclic freezing–thawing, hence the non-standard period between the preparation of the specimens and their testing.

Of note is the very large increase in strength between 28 and 74 days for almost all series of concrete with impregnated aggregate. After 28 days, only two series with impregnated aggregate achieved a strength higher than 1 MPa, and in the case of one series, the strength was close to this value (0.98 MPa). All these series were impregnated with citric acid and water glass. However, these results still remained significantly lower than the tensile splitting strengths of the reference series, which exceeded 4 MPa (NI4) and 3 MPa (NI5), respectively.

After 74 days, the tensile splitting strength developed in a much more balanced manner. While the reference series recorded only a slight increase (with a greater increase in the NI5 series), the increase in the series with impregnated aggregate was at least doubled. In the case of the CL4, CL5 and GC5 series, it was even much greater. In the group of concrete series with w/c = 0.45, the strength of the GC4 series even slightly exceeded the value obtained for the reference series. It is also worth noting the CL4 series, which, after 74 days, obtained a significantly higher strength value than the CG4 series, while after 28 days the situation was the opposite, with a difference of almost three times. Similarly, in the group of concrete series with w/c = 0.55, the greatest increase in strength was recorded by the series with aggregate impregnated with citric acid and lime milk (CL5), although the difference in strength values compared to the CG5 series was small. Within this group, the reference series achieved the highest strength.

In contrast, irrespective of the w/c value and the test date, the series with aggregate impregnated with lime milk and citric acid obtained very low strengths. This was undoubtedly influenced by the order of application of the impregnation solutions. After the second impregnation step, a layer of citric acid-saturated old mortar remained on the aggregate grains, which had already been detached from the aggregate but not removed from it. This layer prevented good adhesion between the aggregate and the new mortar, a fact that the strength test mercilessly exposed.

### 4.3. Results of Sorptivity and Water Absorbability Tests

**Figure 5:**
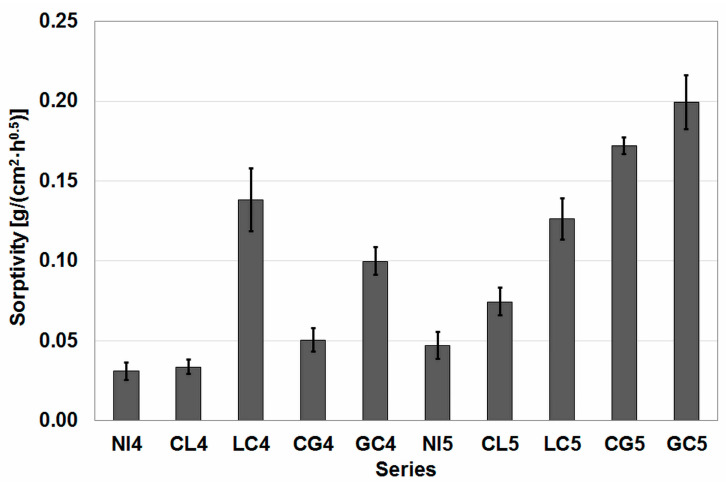
Results of the sorptivity test.

**Figure 6:**
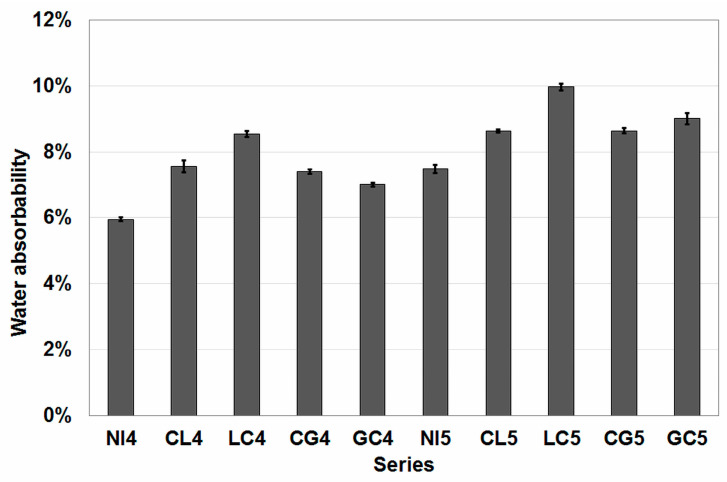
Results of the water absorbability test.

The results of the sorptivity test are shown in Figure 5, and the results of the water absorbability test are shown in Figure 6. The results of these two tests are worth analyzing together, as they concern related phenomena for water transport in concrete.

An analysis of the sorptivity results indicated that this parameter for concrete with impregnated aggregate had the opposite trend to its strength parameters. The w/c ratio had a certain influence on the formation of these trends. In the group of series with a lower value of this coefficient, the highest value of sorptivity was obtained for the LC4 series, and this value was at the same time higher than for the three series of concrete with a higher w/c ratio, including the concrete of the LC5 series, which was in the middle of the range in its group of series. If we exclude the two series that obtained extremely low results in the strength tests, then, after ranking the others according to increasing values of sorptivity, we obtained the same sequence in both groups of concrete series.

Thus, the lowest sorptivity value was obtained for the reference series, followed by series impregnated first with citric acid and then with lime milk (CL4 and CL5, respectively). Next in order are the series impregnated with citric acid and glasswater (CG4 and CG5). The GC4 and GC5 series close the ranks.

The sequence was different for the results of the water absorbability test. The lowest water absorption was recorded for the reference series and the highest for the series in which the aggregate was first impregnated with lime milk and then with citric acid. The series in which citric acid was the first of the impregnants achieved similar water absorption values with the same w/c value. In contrast, the effect of w/c was marked for the series with aggregates impregnated first with glasswater and then with citric acid. At w/c = 0.45, this resulted in the second lowest absorption value, and at w/c = 0.55 in the second highest. In addition, the series with the higher w/c obtained a higher water absorption compared to the corresponding series with a lower value of this parameter.

The difference in pore structure may explain the differences in the trends of the two parameters studied. In the study of sorptivity, capillary pores play a key role. Higher sorptivity with lower absorbability is a strong clue pointing to a higher proportion of capillary pores in the open pore structure. However, mercury intrusion porosimetry (MIP) studies would be required to verify this hypothesis.

### 4.4. Results of Torrent Air Permeability Test

**Figure 7:**
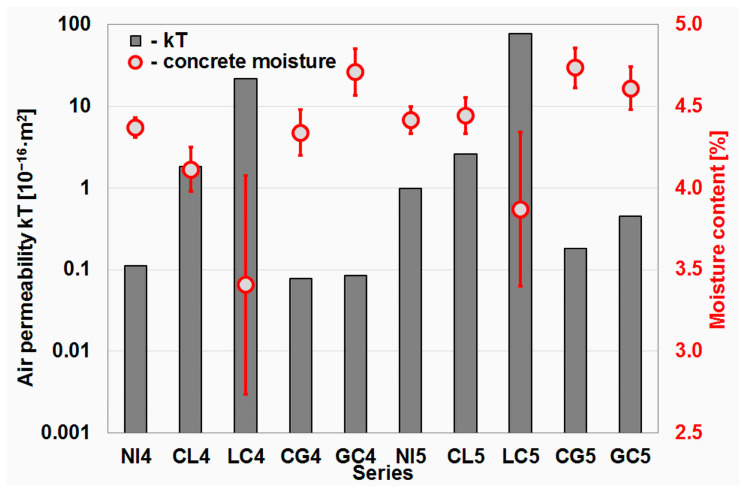
Results of Torrent air permeability test.

The results of the Torrent air permeability test are shown in Figure 7 together with the moisture value that the concrete had at the time of the test. As all the specimens were treated in the same way up to the time of the test, the concrete moisture value can be a valuable guide in interpreting the results of the air permeability test.

Of the durability parameters analyzed so far, the air permeability of the concrete apparently showed that aggregate impregnation has the potential to improve at least some concrete parameters. Of the eight series of concrete with impregnated aggregate, four, i.e., half, obtained lower air permeability values in the tests than the corresponding reference series. These are the series in which citric acid and glasswater were used for impregnation. This is particularly evident in the case of the concrete series with w/c = 0.55. In this group of series, it was also more evident that the order of impregnation matters, as lower permeability was obtained in the study of concrete impregnated first with glasswater and then with citric acid. In the case of a series with a lower w/c value, the difference was small. However, attention should be paid to the moisture values of the concrete at the time of testing. For the GC4 series, it was significantly higher than for the CG4 series (4.71% and 4.34%, respectively). Since the air permeability of the concrete increases as the moisture content of the material decreases [48], it can be assumed that at the same moisture content level, the difference between these series would be more pronounced.

The relationship between moisture content and the permeability of concrete is twofold. On the one hand, as mentioned above, higher concrete moisture content results in lower permeability. This is due to the greater filling of the pores with water and the associated greater resistance that the material places on the flowing air. On the other hand, the rate of moisture loss through the concrete correlates positively with the permeability value. This correlation can be seen in Figure 7, which is due to the fact that the specimens were first saturated with water before being tested and then subjected to the same drying procedure. Those whose moisture content dropped to lower values after this procedure in the permeability tests generally achieved higher results. However, there is no direct correlation between the two values, although their mutual relationship is worth examining in a dedicated study.

### 4.5. Influence of Freeze–Thaw Cycles on Compressive and Tensile Splitting Strength

**Figure 8:**
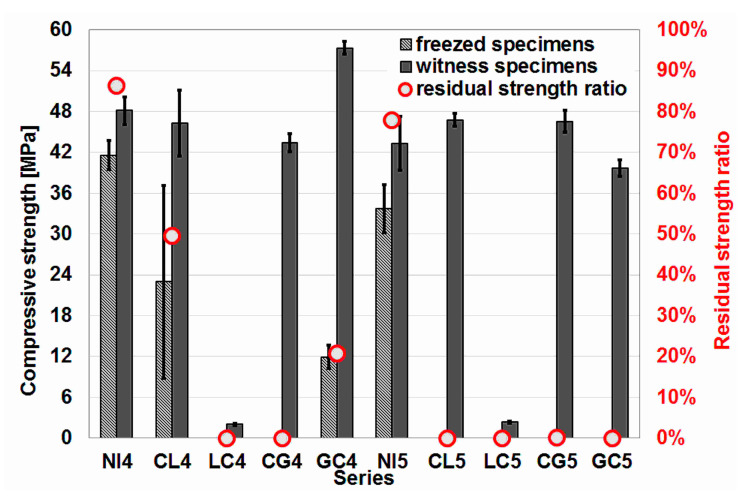
Results of the freeze–thaw resistance test.

**Figure 9:**
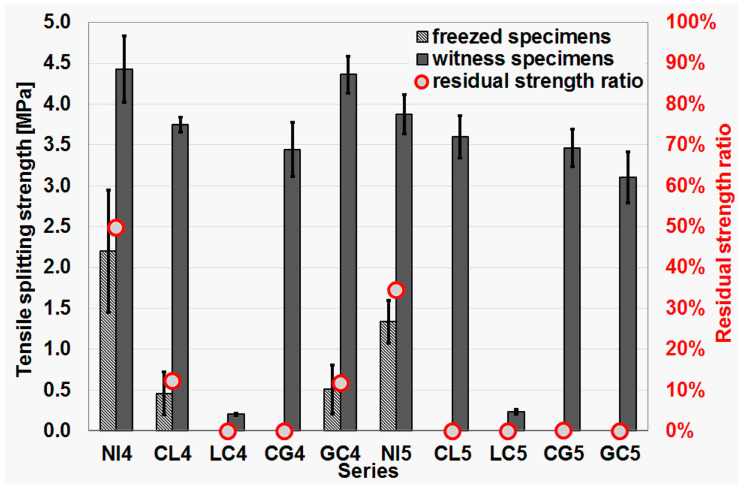
Results of the tensile splitting strength test after freeze–thaw cycles.

Results of the freeze–thaw resistance test and the test of reduction of the tensile splitting strength after freeze–thaw cycles are presented in Figure 8 and Figure 9, respectively.

After 100 freeze–thaw cycles, only specimens from four series were suitable for strength testing. In addition to the two reference series, these were two concrete series with w/c = 0.45: a series with aggregate impregnated first with citric acid and then with lime milk, and a series with aggregate impregnated first with glasswater and then with citric acid. The smallest decrease in strength among the four series was recorded for the NI4 reference series, and only in this case was the decrease less than 20%. In the case of the series with impregnated aggregate, the decrease was twofold and fourfold.

The decrease in splitting tensile strength after 100 freeze–thaw cycles was significantly greater. In the case of the NI4 reference series, the decrease was the smallest but still amounted to more than 50%. In contrast, the series with impregnated aggregate only reached just over 10% of the strength determined on the specimens not subjected to cyclic freeze–thawing tested at the same time. 

Despite the small number of examples (4 concrete series), it can be concluded that the splitting tensile strength of concrete is a parameter that is much more sensitive to the influence of cyclic freeze–thaw.

## 5. Conclusions

The obtained results allowed us to formulate the following conclusions.

- Due to the strong retarding effect of citric acid on the setting and hardening of the cement, the negative effect of impregnation on the strength parameters of the concrete with impregnated aggregate was very evident after 28 days, while after 74 days, the strength values of the concrete decreased only slightly compared to the reference series. In three cases, the compressive strength at that time proved to be even higher.
- The sorptivity of concrete with impregnated aggregate was generally higher, but the increase of its value depended strongly on the value of the w/c ratio. For the higher w/c ratio, the increase in sorptivity was significantly greater.
- Water absorbability of concrete with the impregnated aggregate was higher, but citric acid combined with glasswater sealed the aggregate more efficiently than the other combination of impregnants.
- The air permeability of concrete made with aggregate impregnated with water glass and citric acid (in any sequence) was found to be lower than that of the reference concrete regardless of the value of the w/c ratio. Aggregate impregnation carried out with citric acid and lime milk resulted in an increase in air permeability of the concrete.
- The use of impregnated aggregate strongly negatively affected the resistance of the concrete for cycling freezing.
- The sequence of application of the impregnating solutions had a very strong influence on the results, with generally more favorable results being obtained when the citric acid solution was applied first. Glasswater performed better as the second impregnant, with the sequence of application having a smaller effect on the results obtained.

To sum up, analyzing both the course of the impregnation process itself and the results obtained, it can be concluded that in order to reduce the negative effect of citric acid, it would be advisable to use it in a lower concentration or to introduce an additional operation of rinsing the aggregate after impregnation with the acid solution. Both of these modifications could be the subject of further research, as despite the generally poorer results obtained for concrete with impregnated aggregates, these treatments appear to have the potential to improve the performance of concrete with such aggregates.

## Figures and Tables

**Table 1:** Mix composition of the prepared concrete series (in kg/m^3^).

Component	NI4	CL4	LC4	CG4	GC4	NI5	CL5	LC5	CG5	GC5
Cement CEM I 42.5R	325	325	325	325	325	325	325	325	325	325
River sand (0–2 mm)	676	676	676	676	676	646	646	646	646	646
RCA (4–16 mm)	1137	1137	1137	1137	1137	1086	1086	1086	1086	1086
Water	146	146	146	146	146	179	179	179	179	179
Plasticizer	1.63	3.25	3.25	4.06	3.25	0.00	1.63	1.63	1.63	0.81
w/c ratio	0.45	0.45	0.45	0.45	0.45	0.55	0.55	0.55	0.55	0.55

**Table 2:** Results of tests of mechanical and durability parameters (with measures of the variation).

Parameter	NI4	CL4	LC4	CG4	GC4	NI5	CL5	LC5	CG5	GC5
Compressive strength after 28 days (100 mm cubes) [MPa]	56.2 (2.9)	8.21 (0.49)	1.53 (0.05)	17.0 (1.9)	16.4 (0.7)	40.1 (1.4)	1.57 (0.15)	0.58 (0.06)	11.1 (0.7)	1.59 (0.21)
Compressive strength after 37 weeks (150 mm cubes) [MPa]	70.8 ± 0.5	48.1 ± 5.2	6.31 ± 0.36	68.2 ± 5.1	63.7 ± 0.3	54.8 ± 4.0	35.9 ± 3.3	5.06 ± 2.28	49.9 ± 0.7	43.9 ± 3.1
Tensile splitting strength after 28 days [MPa]	4.37 (0.43)	0.66 (0.18)	0.09 (0.01)	1.72 (0.14)	1.62 (0.08)	3.60 (0.14)	0.17 (0.02)	0.05 (0.00)	0.98 (0.04)	0.15 (0.01)
Water absorbability [%]	5.95 (0.05)	7.55 (0.18)	8.53 (0.09)	7.41 (0.06)	7.01 (0.06)	7.48 (0.12)	8.62 (0.05)	9.97 (0.10)	8.64 (0.09)	9.01 (0.17)
Sorptivity [mg/(cm^2^·h^0.5^)]	30.9 (5.2)	33.6 (4.6)	138 (20)	50.5 (7.4)	99.9 (8.7)	47.2 (8.4)	74.4 (8.7)	126 (13)	172 (5)	199 (17)
Torrent air permeability [10^−16^·m^2^]	0.11 {1.41}	1.80 {1.79}	21.5 {2.13}	0.08 {1.14}	0.09 {1.31}	0.98 {1.61}	2.63 {1.63}	>77.7 ^1^ {-- ^1^}	0.18 {1.22}	0.45 {1.51}
Concrete moisture at permeability test [%]	4.37 (0.06)	4.11 (0.13)	3.41 (0.67)	4.34 (0.14)	4.71 (0.14)	4.42 (0.08)	4.44 (0.11)	3.87 (0.47)	4.73 (0.12)	4.61 (0.13)

^1^ The lower limit of the geometric mean value was calculated assuming a value of 100.1 × 10^−16^ m^2^ for 6 of the 8 measurements that exceeded the upper measurement range of the device (100 × 10^−16^ m^2^); the value of the average geometric deviation was not calculated.

**Table 3:** Results of freeze–thaw resistance test (compressive and tensile splitting strength).

Parameter	NI4	CL4	LC4	CG4	GC4	NI5	CL5	LC5	CG5	GC5
Compressive strength of the frozen specimens (MPa)	40.5 (1.1)	22.0 (13.3)	--	--	12.9 (1.5)	36.4 (3.3)	--	--	--	--
Compressive strength of the witness specimens (MPa)	48.1 (2.1)	46.3 (4.9)	2.03 (0.18)	43.4 (1.4)	57.3 (0.9)	43.3 (4.0)	46.8 (1.0)	2.34 (0.14)	46.6 (1.6)	39.7 (1.2)
Compressive strength loss (%)	15.9	52.5	--	--	77.5	16.0	--	--	--	--
Tensile splitting strength of the frozen specimens (MPa)	2.20 (0.75)	0.46 (0.27)	--	--	0.51 (0.30)	1.34 (0.26)	--	--	--	--
Tensile splitting strength of the witness specimens (MPa)	4.43 (0.41)	3.75 (0.09)	0.21 (0.02)	3.44 (0.33)	4.36 (0.23)	3.88 (0.24)	3.60 (0.26)	0.24 (0.03)	3.46 (0.23)	3.11 (0.32)
Tensile split. strength loss (%)	50.3	87.7	--	--	88.3	65.5	--	--	--	--

## Data Availability

Data available on demand.
